# Treatment of Headache in the Elderly

**DOI:** 10.1007/s11940-012-0205-6

**Published:** 2012-10-11

**Authors:** Linda A. Hershey, Edward M. Bednarczyk

**Affiliations:** 1 Department of Neurology, University of Oklahoma, 711 Stanton L. Young Blvd, Suite #215, Oklahoma City, OK 73104-5021 USA; 2 Department of Pharmacy Practice, University at Buffalo, 222 Kapoor Hall, Buffalo, NY 14260 USA

**Keywords:** Cluster, Headache, Treatment, Elderly, Divalproex, Hydroxyzine, Hypnic, Lithium, Magnesium, Medication overuse, Metoclopramide, Metoprolol, Migraine accompaniment, Naproxen, Propranolol, Sleep apnea, Subarachnoid hemorrhage, Temporal arteritis, Tension, Thunderclap, Topiramate, Trigeminal neuralgia

## Abstract

Most primary headaches in the elderly are similar to those in younger patients (tension, migraine, and cluster), but there are some differences, such as late-life migraine accompaniments and hypnic headaches. Although migraine in younger persons usually presents with headache, migraine in older persons may initially appear with visual or sensory phenomena, instead of headache (“migraine accompaniments”). Hypnic headaches awaken patients from sleep, are short-lived, and occur only in the elderly. The probability of secondary headache increases steadily with age. Secondary headaches include those associated with temporal arteritis, trigeminal neuralgia, sleep apnea, post- herpetic neuralgia, cervical spondylosis, subarachnoid hemorrhage, intracerebral hemorrhage, intracranial neoplasm, and post-concussive syndrome. Certain rescue treatments for migraine headache in younger individuals (triptans or dihydroergotamine, for example) should not be used in elderly patients because of the risk of coronary artery disease. Naproxen and hydroxyzine are commonly used oral rescue therapies for older adults who have migraine or tension headaches. Intravenous magnesium, valproic acid, and metoclopramide are all effective rescue therapies for severe headaches in the emergency room setting. Some effective prophylactic agents for migraine in younger patients (amitriptyline and doxepin) are not usually recommended for older individuals because of the risks of cognitive impairment, urinary retention, and cardiac arrhythmia. For these reasons, the recommended oral preventive agents for migraine in older adults include divalproex sodium, topiramate, metoprolol, and propranolol. Oral agents that can prevent hypnic headaches include caffeine and lithium. Cough headaches respond to indomethacin or acetazolamide.

## Introduction

Headache (HA) continues to be one of the most frequent complaints in neurology offices today. The prevalence of primary HA declines steadily after the age of 40, but secondary HA is more likely to be seen in the elderly [1–3]. In one series, 15 % of elderly patients who presented de novo with HA had a serious treatable disorder, such as subarachnoid hemorrhage, temporal arteritis, trigeminal neuralgia, or intracranial hemorrhage, whereas only 1.6 % of those under 65 had a similarly serious condition [3]. Hospital admissions for intracerebral hemorrhage have increased by 18 % in the past 10 years, probably as a reflection of older individuals whose hypertension is not adequately controlled [4]. Other contributing factors include the increased use of anticoagulant drugs and the increased prevalence of cerebral amyloid angiopathy [4]. Other secondary HAs include those caused by sleep apnea [5]. A recent study showed that HA improvement could be seen in 49 % of sleep apnea patients who were treated with continuous positive airway pressure (CPAP).

HA in the elderly can be divided into primary and secondary types. The most common primary HA types in the elderly are tension, migraine, late-life migraine accompaniments, cluster, and hypnic HA [1, 2, 18]. Tension HA is the most common primary HA type in the elderly, according to one door-to-door survey of 833 elderly subjects in Italy [1]. The 1-year prevalence rate for tension HA in that series was 44.5 %, compared with 11.0 % for migraine HA, 4.4 % for chronic daily HA, and 2.2 % for symptomatic HA. The American Migraine Study [2] showed that the prevalence of migraine HA was 25 % in 50-year-old women, whereas it was only 10 % in 70-year-old women. For 50-year-old men, the prevalence of migraine HA was about 8 %, and this fell to about 5 % by the age of 70 years.

Chronic daily HA is an important group that includes transformed migraine, chronic tension HA, and hemicrania continua [6]. In a large epidemiologic study in Spain, the prevalence of chronic daily HA in older women (>60) was 11.3 % (a much higher prevalence rate than that seen in women in general, which was 8.7 %). Overuse of symptomatic medications was reported in 19 % of the chronic tension type HA patients in that series (acetaminophen, aspirin, codeine, and caffeine). Even more of the transformed migraine patients in that series (31 %) overused drugs (ergotamine, caffeine, and barbiturates). Several of the patients in that series had been placed on chronic analgesics for non-HA indications, such as low back pain, demonstrating that HA-prone patients are vulnerable to developing rebound HA, even though they are being medicated for non-HA disorders. According to International Headache Society (IHS), neither chronic migraine, nor medication-overuse HA can be diagnosed with confidence until medication has been withdrawn. If the medication-overuse HA diagnosis is correct, improvement should be expected within 2 months [7].

Medication may be seen as the cause of HA in as many as 8 % of patients who present with a HA complaint [7]. Nitroglycerine, for example, is a vasodilator that can trigger a nonspecific HA in non-migraineurs and a migraine-like HA in those who have a history of migraine [8]. Nifedipine and dipyridamole are central vasodilators that are known to cause HA [8]. Selective serotonin reuptake inhibitors, such as fluoxetine, paroxetine, sertraline, citalopram, and escitalopram have been commonly associated with drug-induced HA [9]. Venlafaxine, an inhibitor of serotonin, norepinephrine, and dopamine reuptake, has also been associated with drug-induced HA [9].

Migraine in younger persons usually presents with a HA or nausea complaint, whereas older individuals with migraine may develop other symptoms at onset, such as scintillating scotoma, traveling paresthesias, homonymous field defects, or speech disturbances [10, 11]. Typical features of these late onset migraine accompaniments are the buildup and spread of visual scintillations, spreading of the paresthesias from the hand to the face, and a progression from one accompaniment to the other. The total duration of migraine symptoms is usually 15–30 min, whereas transient ischemic attacks (TIA) in the vertebrobasilar system are shorter lasting (less than 15 min). HA is present in about 50 % of patients who have migraine accompaniments, and a personal history and family history of migraine are often obtained from the patient. Regional cerebral blood flow patterns in those who have migraine accompaniments are more likely to resemble age-matched controls than those who have posterior circulation TIA [12]. Posterior circulation TIAs may have HA as one of the prodromal symptoms that precede stroke by days or weeks [13]. In patients with basilar artery occlusion, the most common predictive symptoms are vertigo, nausea, and HA [14]. These symptoms may occur as early as 2 weeks or more prior to the onset of stroke.

A sudden, severe HA with maximum onset within 1 min and without evidence of subarachnoid hemorrhage (“thunderclap HA”) can occur in association with any of the primary HA disorders, but other causes also need to be considered [15]. Thunderclap HA patients need a thorough set of neuro-imaging studies to rule out other possible diagnoses, such as an aneurysm, or cerebral venous thrombosis. Primary thunderclap HA usually reaches its maximum intensity at 30 min and can last hours to weeks. They can be triggered by exercise, diving into cold water, walking into cold wind, or coughing.

Cough HA (the primary, benign type) is of sudden onset and lasts for 1 second to 30 min. It is usually bilateral and affects a predominance of male patients over the age of 40 years [16]. Cough HA begins significantly later than benign exertional HA, or benign vascular sexual HA. Several cases of symptomatic cough HA were related to Chiari type I malformation in the study of Pascual et al. [16], so these patients all need to have MRI scans with sagittal views to make the correct diagnosis.

Cluster HA, the most severe of all the primary HA disorders, can begin as late as 83 years of age [17]. It is characterized by unilateral pain, ipsilateral autonomic features, and motor restlessness. Inheritance with cluster HA is autosomal dominant, but there is low penetrance in some families. In certain families, one patient may have cluster HA, while another person may have the autonomic symptoms without the HA. Genetic anticipation has been seen in cluster HA families, so that the children of the probands have earlier onset of disease, and the symptoms become worse with each successive generation.

Hypnic HA awakens a patient from sleep and almost always begins after the age of 60 [18]. The pain is usually dull and diffuse and lasts for 30–60 min at a time. There are no autonomic symptoms to suggest the diagnosis of cluster HA. The pain of hypnic HA does not have lightening-like qualities to suggest the diagnosis of trigeminal neuralgia. Hypnic HA can usually be prevented by taking coffee (1–2 cups) or lithium (150–600 mg) prior to bedtime [18].

Cardiac ischemia can rarely be associated with a unilateral or bilateral HA that is brought on by exercise and is relieved by rest [19–21]. When one obtains this history, a prompt referral to a cardiologist or to the emergency room is urgently needed.

The choice of HA medication should be based on the type of HA that is being treated, the drug’s efficacy, side effects, and costs. Certain HA drugs, such as the triptans, cannot be used in the older patients with established cardiovascular disease, since the risk of vasospasm is too high [22].

## Treatment

### Weight modification

Compared with those with normal body mass index (BMI), those with morbid obesity have a greater than two-fold increased odds of migraine [23]. Six months after bariatric surgery, the monthly HA frequency declined significantly [24, 25].

### Exercise

Some randomized trials have shown that migraine patients benefit from regular exercise, when it is offered as an alternative to relaxation therapy, or medication [26].

### Intravenous agent for acute HA therapy: magnesium sulfate

Intravenous magnesium (2 g over 10 min) has been shown to be comparable to IV metoclopramide (10 mg over 10 min) for acute migraine attacks [27]. This drug plays a role in regulating vascular tone, which is thought to be important in migraine pathophysiology.

**Standard dosage**: 2 grams in 100 mL of normal saline/infused over 10 min.
**Contraindication**: Known adverse reaction to MgSO4; altered mental status; fever; renal or cardiac disease.
**Main side effects**: Altered mental state; hypotension; arrhythmia.

### Intravenous agent for acute HA therapy: metoclopramide

In one double-blind study, IV metoclopramide had an efficacy of 67 % at 1 h, compared with 19 % with placebo [28]. In another trial, IV metoclopramide was dosed up to 20 mg four times over 24 h without causing complaints of chest pain (SQ sumatriptan was the comparison group). Efficacy in that study was 59 % at 2 h for metoclopramide, compared with 35 % for SQ sumatriptan [29].

**Standard dosage**: 10 mg in 2 mL of saline over 10 min (up to 80 mg in 24 h).
**Contraindication**: Parkinson’s disease or Parkinson-plus syndrome.
**Main side effect**: Motor restlessness, weakness, dizziness, drowsiness.

### Intravenous agent for acute HA therapy: ketorolac

Intravenous ketorolac (30 mg over 30 min) is a cyclooxygenase inhibitor that can reverse peripheral sensitization by inhibiting the neuroinflammatory cascade. It is more efficacious (60 %) than sumatritan nasal spray for treating acute migraine HA [30]. Consider using a lower dose in older persons especially in someone with reduced renal function.

**Standard dosage**: 30 mg in 50 mL of saline or D5W over 30 min.
**Contraindication**: Patients already on anticonvulsants, antidepressants, or tranquilizers; history of allergic reactions to NSAIDs; pregnancy, use of alcohol.
**Main side effects**: Drowsiness, dizziness, nausea, and stomach pain.

### Intravenous agent for acute HA therapy: valproic acid

Valproic acid increases gamma-aminobutyric acid levels in the brain, which leads to inhibition of the trigeminal nerve activation. Intravenous valproic acid (800 mg) is comparable to IV lysine-acetylsalicylic acid (1000 mg) at 24 h in acute migraine [31].

**Standard dosage**: 500–1000 mg in 100 cc of normal saline infused over 30 min.
**Contraindication**: Known allergy to valproic acid, pregnancy, or liver disease.
**Main side effects**: Nausea, sedation, diarrhea.

### Oral agent for HA prophylaxis: divalproex sodium

There is strong, consistent support for the use of divalproex sodium for prevention of migraine HA [32, 33]. There is level A evidence for efficacy, according to AAN Guidelines [34••]. It is well tolerated in all age groups.

**Standard dosage**: 500–1500 mg/d.
**Contraindication**: Liver disease.
**Main side effects**: Weight gain with long-term use; teratogenic risk in young patients; pancreatitis and liver failure in rare cases.

### Oral agent for HA prophylaxis: topiramate

Another anticonvulsant with level A evidence for efficacy in migraine prophylaxis is topiramate. It is more effective than either placebo or lamotrigine [35]; it is equally effective, compared with propranolol [36] and sodium valproate [37].

**Standard dosage**: 25–200 mg/d (start 25 mg qhs for the first week, then use 25 mg bid for the first month; if excessive daytime sedation develops, use 50 mg qhs).
**Contraindication**: Kidney stones.
**Main side effects**: Paresthesias, sleepiness, mild nausea, anorexia, weight loss, word-finding difficulties, and kidney stones (patients need to be encouraged to drink plenty of water to prevent the kidney stones).

### Oral agent for HA prophylaxis: metoprolol

Metoprolol (200 mg/d) is more effective (45 %) than aspirin (30 %) (300 mg/d) in reducing HA frequency in patients with migraine [38].

**Standard dosage**: 100–200 mg/d.
**Contraindication**: Chronic lung disease, asthma, congestive heart failure.
**Main side effects**: Dizziness, fatigue, impotence, wheezing.

### Oral agent for HA prophylaxis: propranolol

Propranolol (80 mg/d) is more effective than placebo in reducing HA frequency and severity [39].

**Standard dosage**: 40–80 mg/d.
**Contraindication**: Chronic lung disease, asthma, congestive heart failure.
**Main side effects**: Dizziness, fatigue, impotence, wheezing.
